# Effect of Genotype on the Response to Diet in Cardiovascular Disease—A Scoping Review

**DOI:** 10.3390/healthcare12222292

**Published:** 2024-11-16

**Authors:** Génesis K. González-Quijano, Guadalupe León-Reyes, Eliane Lopes Rosado, J. Alfredo Martínez, Daniel A. de Luis, Omar Ramos-Lopez, María Elizabeth Tejero

**Affiliations:** 1 Laboratory of Nutrigenetics and Nutrigenomics, Instituto Nacional de Medicina Genómica (INMEGEN), Mexico City 14610, CDMX, Mexico; ggonzalez@inmegen.edu.mx (G.K.G.-Q.); greyes@inmegen.gob.mx (G.L.-R.); 2 Consejo Nacional de Humanidades, Ciencia y Tecnología-Instituto Nacional de Medicina Genómica (CONAHCYT-INMEGEN), National Council for Humanities, Science and Technology, Mexico City 03940, CDMX, Mexico; 3 Nutrition and Dietetics Department, Institute of Nutrition Josué de Castro, Federal University of Rio de Janeiro, 373 Carlos Chagas Filho Avenue, Sector J, 2nd Floor, University City, Rio de Janeiro 21941-902, Brazil; elianerosado@nutricao.ufrj.br; 4 Precision Nutrition and Cardiometabolic Health, IMDEA-Food Institute (Madrid Institute for Advanced Studies), Campus of International Excellence (CEI) UAM+CSIC, 28049 Madrid, Spain; jalfredo.martinez@imdea.org; 5 Centro de Medicina y Endocrinología, Universidad de Valladolid, 47011 Valladolid, Spain; 6 Centro de Investigación Biomédica en Red de la Fisiopatología de la Obesidad y Nutrición (CIBERobn), Instituto de Salud Carlos III, 28029 Madrid, Spain; 7 Center of Investigation of Endocrinology and Nutrition, Department of Endocrinology and Investigation, Medicine School, Hospital Clinico Universitario, University of Valladolid, 47011 Valladolid, Spain; dluisro@saludcastillayleon.es; 8 Medicine and Psychology School, Autonomous University of Baja California, Tijuana 22390, BC, Mexico

**Keywords:** nutrigenetics, intervention, obesity, cardiovascular disease, genotype

## Abstract

Background/Objectives: Nutrigenetics investigates the role of genetic variants that contribute to the inter-individual variation in response to food intake. Risk factors for cardiovascular disease (CVD) are influenced by the complex interplay of genetic and environmental factors, including the diet. The aim of this scoping review is to analyze the literature on the effect of genotypes on the response to dietary interventions for the treatment of CVD risk factors. Methods: A literature search was conducted in MEDLINE to identify published articles fulfilling the inclusion criteria. Studies published in English between 2014 and 2024 were selected. Data were extracted according to the population, intervention, comparison, and outcome (PICO) format. Results: Forty-eight studies met the inclusion criteria. The studies differed in design, intervention characteristics, tested genotypes, and ancestry. The most frequently analyzed variants were single-nucleotide polymorphisms (SNPs) in genes associated with lipid metabolism, inflammation, and energy balance, among others. The interventions tested the effects of different dietary patterns, diets modified in macronutrient content and types of fat, natural and processed foods, nutraceuticals, and nutrient supplements. Common *APOE* variants were the most analyzed genotypes showing significant interactions with different dietary interventions affecting blood lipids. Other genotypes found in pathways involving folic acid, lipid metabolism and transport have shown interactions with diverse dietary components across studies. Conclusions: Gene–diet interactions are observed in multiple dietary interventions. Replication of findings of nutrigenetic studies is required across different populations. The response to dietary treatments modifies CVD-related risk factors and shows variation associated with genotypes.

## 1. Introduction

Chronic, non-communicable diseases are the main causes of disease and mortality in adults [1]. Data from the World Health Organization showed that cardiovascular diseases (CVDs) are the leading cause of death globally, taking an estimated 17.9 million lives each year [2]. Obesity is a multifactorial disease and a risk factor for other diseases such as CVD [3]. CVD is a group of disorders of the heart and blood vessels and includes coronary heart disease, cerebrovascular disease, rheumatic heart disease and other conditions, and it is the leading cause of morbidity and mortality in adults worldwide [2]. Dietary habits influence diverse cardiometabolic risk factors including blood lipids, obesity, hypertension, glucose–insulin homeostasis, lipoprotein concentrations and function, oxidative stress, inflammation, and endothelial health, among others [4]. These phenotypes are well-established risk factors for CVD and other diseases that exhibit high inter-individual variability in response to diet. This variation is explained, to some extent, by variants across multiple genes [5,6].

In this sense, nutrigenetics refers to the study of the interaction between genetics and nutrition, investigating the effect of genetic variation on the response to diet, or its components [7,8,9]. There is wide evidence that genetic variation, particularly single-nucleotide polymorphisms (SNPs), has a significant influence on the absorption, synthesis, utilization, and transport of substances present in food or their metabolites, with an impact on health. Some of these effects were identified in cross-sectional studies showing differences in phenotypes (i.e., body mass index (BMI), high-density lipoprotein cholesterol (HDL-C)) as an outcome of the interaction between nutrients and gene variants. A classic example of gene–diet interaction is the effect of the T allele of the -1131 T > C in *APOA5* variant (rs662799) and dietary fat intake on BMI [10] and the capacity to clear chylomicron triglycerides (TGs) or hydrolyze TGs [11]. Some of these effects have been replicated across populations [12,13,14]. Other factors that contribute to diversity are epigenetic mechanisms and the activity of the gut microbiome, that interact with other environmental, social and demographic individual characteristics [8]. Later studies are clinical trials that identified numerous significant gene–diet interactions [14,15], and more recently, researchers developed genetic risk scores (GRSs) that integrate the weighted effect of a group of variants in order to predict the response to an intervention [16].

The characterization of gene–diet interactions influencing CVD provide information for developing more efficient tools for promoting health and wellness and is required for personalized nutrition and precision healthcare.

The aim of the present review is to map and describe the scientific literature on the effect of genotypes or groups of them (i.e., GRS) on the response to dietary interventions for the treatment of risk factors for CVD.

## 2. Materials and Methods

This review was based on the international guide Preferred Reporting Items for Systematic review and Meta-Analyses extension for Scoping Reviews (PRISMA-ScR) [17]. The research question was as follows: What is the effect of genetic variation (SNPs) on changes in phenotypes associated with CVD, in response to dietary interventions? A search was conducted in MEDLINE using the following algorithm: (diet OR gene-diet OR dietary OR nutrient OR nutraceutic OR functional food OR bioactive compound) AND (cholesterol OR blood lipids OR hypertension OR cardiovascular OR coronary) AND (SNP OR polymorphism OR genetic variant) NOT (children) NOT (review), using the filter Adults. The search considered publications between 2014 and 2024. The date for the last search was 27 August 2024. The criteria for study eligibility were as follows: published studies, in English language, testing a dietary intervention conducted in adults (>18 y), using a nutrigenomic approach (studies testing the effect of a genotype, or group of them, on the response to an intervention with a dietary pattern, modified diet or compound from food origin); the search was limited to effects of SNPs and included studies on individual or collective SNPs’ effects. The dietary intervention included modified diets, dietary patterns, consumption of specific foods according to an indication, nutraceuticals or nutrient supplements, regardless of duration, with the exception of acute or single-dose effects. Cardiovascular disease-related risk factors included total cholesterol, low-density lipoprotein cholesterol (LDL-C), HDL-C, TG, lipoproteins a (Lp-a), blood pressure (BP), and homocysteine blood concentrations.

Exclusion criteria were as follows: studies evaluating the effect of a single dose of any meal, nutrient, food, or bioactive compound, reviews, and interventions that included other components different from diet or diet-derived compounds, such as exercise or other lifestyle changes, medication, etc. Records retrieved were screened and pre-selected based on the titles and abstracts. Examination of the full document was conducted according to eligibility criteria. The data extraction followed a population, intervention, comparison, and outcome (PICO) format. The included information was as follows: characteristics of the studied population (sample size, age, BMI, diagnosis, and ancestry), intervention (description of treatment, dose, time) comparison (genotypes of GRS, and in some cases, different diets), and outcome (effects of genotypes on the main phenotype/s).

Additionally, this study adhered to the PRISMA (Preferred Reporting Items for Systematic Reviews and Meta-Analyses extension for Scoping Reviews; PRISMA-ScR) guidelines (Supplementary Materials). This protocol was registered in Open Science Frame work (OSF; https://osf.io/ub2rd, accessed on 12 November 2024) doi: 10.17605/OSF.IO/UB2RD.

## 3. Results

**Figure 1:**
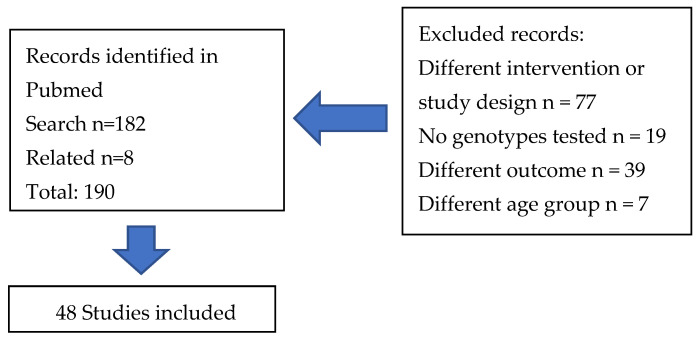
Flow diagram of the study selection.

The search on nutrigenetic studies on CVD and related phenotypes retrieved 190 articles (Figure 1). The search identified original studies published between January 2014 and July 2024. This review selected intervention studies in order to investigate cause and effect relationships. A group of 48 studies that fulfilled the inclusion criteria were considered for data extraction and classified into groups according to the intervention characteristics (Table 1, Table 2, Table 3, Table 4, Table 5 and Table 6). The studies were conducted in participants with diseases such as obesity, different forms of dyslipidemia and other CVD risk factors, and other conditions, and healthy adults. The most investigated group is Caucasians in Europe and North America. As observed, a small number of studies have been conducted in other populations across the world.

The sample size ranged between 36 and 7170 participants, and there was a wide variation in duration, characteristics of the interventions, and study designs, which makes it difficult to compare or integrate results. The selected investigations analyzed the effect of a single variant, the separated or combined effects of two or more variants, and polygenic scores. The most frequently analyzed SNPs are located at genes involved in lipid metabolism (apoproteins, enzymes, and other proteins involved in cholesterol and fatty acid metabolism, and receptors, among other functional proteins), inflammation (*TNFα*, *ADIPOQ*), appetite regulation (*NPY*, *BDNF*), circadian rhythm (*CLOCK*) energy balance (*PPAR*s, *ADBR2*), folic acid metabolism (*MTHFR*, *MTR*, *MTRR*) and other less investigated pathways. Interventions were classified as dietary patterns (n = 11), supplementation with vitamins with mixed or isolated nutrients (n = 3), and diets modified in fat content (n = 15) (this category included studies using fatty acid supplements). Diets modified in macronutrient content (n = 3) were assigned to a different category that investigated the effect of macronutrient composition, fat types and glycemic index, hypocaloric diets (n = 2), and the last category contains studies with interventions different from the previously described, classified as “others” (n = 14). This category included studies using unprocessed foods, such as kiwi, oatmeal, rice, drinks (red wine), salt, processed food (a snack containing cholesterol-lowering compounds), food groups (dairy products), a nutraceutic (artichoke leaf extract), and isolated food compounds (low- and high-molecular-weight β-glucans).

The phenotypes significantly affected by gene–diet interactions were LDL-C, HDL-C and TG, together with other circulating lipid fractions, blood homocysteine, and markers for glucose metabolism and inflammation. Three studies tested interaction effects on BP.

## 4. Discussion

As shown in Table 1, Table 2, Table 3, Table 4, Table 5 and Table 6, the gene–diet interaction affecting CVD risk factors has been extensively investigated using a wide variety of experimental approaches.

### 4.1. Effects of Genotypes

In the case of apolipoprotein E (APOE) variants, the present review identified seven studies showing significant interactions with dietary interventions using different types of fatty acids, phytostanols, and β-glucans [19,21,26,27,48,53,66]. APOE participates in the lipoprotein-mediated lipid transport between organs via the plasma and interstitial fluids. The APOE2, 3 and 4 phenotypes had a significant effect on CVD risk factors and dietary response [48,63]. The study by Rajediran et al., 2021 [19] analyzed the interaction between a group of variants in genes involved in cholesterol metabolism, and only variants in the *APOE* and *ABCA1* genes showed consistent effects across interventions using different types of fat modifying HDL-C, LDL-C, and other CVD-associated risk factors. In the mentioned study, the APOE2 genotype exhibited significantly lower serum LDL-C concentrations than E4 carriers following a diet containing butter as a source for saturated fat, and they had lower serum LDL-C concentrations than E3 carriers following a diet containing PUFAs (*p* = 0.011). A previous study [48] reported a differential response to the substitution of saturated fats (SFAs) by monounsaturated fatty acids (MUFAs) and low glycemic index (GI) carbohydrate in lipids by APOE. Another study [27] showed genotype-dependent effects in the response to diets containing different types of fat. Findings of this investigation showed that APOE rs1064725 and a high-MUFA diet had a significant interaction, and TT homozygotes had significantly lower total cholesterol with this diet compared with a high saturated fatty acid (SFA) diet and ω-6 polyunsaturated fatty acid (PUFA) diet (*p* = 0.003 and *p* = 0.004) (Table 1). A possible mechanism explaining these findings is that a high-MUFA diet could shorten the residence time of very low-density lipoprotein (VLDL) particles in the circulation, increasing the triglyceride-rich lipoprotein clearance rate among TT homozygotes of *APOE* rs1064725. These studies indicate that APOE4 carriers are responsive to dietary MUFAs. In line with these findings, similar genotype-dependent effects were observed after the exposure to diets with different content and type of fat, and less beneficial effects were found in APOE4 carriers [26]. According to Fallaize et al., 2017, APOE4 carriers generally have higher TC and LDL-C levels [57]. The study found that APOE variants modify the decrease in LDL-C after treatment with phytostanols. According to this study, APOE4 had a greater decrease in this cholesterol fraction as compared to APOE3 carriers [63]. In addition, APOE isoforms are associated with different responses to soluble fiber in lowering circulating LDL-C concentrations [67]. There is a controversy on the effect of the APOE isoform on the response to diet and association with risk factors for CVD. It is believed that the isoforms of APOE alter the physical structure of the lipoprotein itself, and E2 isoform reduces the affinity of the particle to bind to LDL receptors (LDL-R), explaining why E2 transport is associated with low levels of LDL-C; however, Minihane et al. (2007) considered that E3 and E4 protein isoforms do not bind differently to the LDL-R [68]. It has been proposed that the E4 isoform has a preferential association with triglyceride-rich lipoproteins (TRLs), resulting in more APOE bound to these particles, causing competition at the LDL-R, and an increase in circulating cholesterol; in addition, the conversion of VLDL into LDL occurs at a faster rate in APOE4 carriers [26].

The G allele of the rs3808607 variant in the gene cytochrome P450, family 7, subfamily A, polypeptide 1 (*CYP7A1*) showed differential effects on LDL-C levels after treatment with phytostanols, with G carriers having a larger decrease (*p* < 0.001) [63]. This gene encodes an enzyme that regulates bile acid biosynthesis and cholesterol homeostasis. The mentioned variant is within the gene promoter region and enhances gene expression, increasing bile acid synthesis [69]. A few studies testing the cholesterol lowering effects of the consumption of oatmeal and its β-glucans and the interaction with the rs3808607 variant have been conducted. The study by Ye M. et al. [53] found a significant interaction between this genotype and oatmeal intake reducing LDL-C. In this study, the TT carriers had a larger reduction in LDL-C as compared with the G allele. A previous study found that CYP7A1 rs3808607-G carriers are more responsive to the cholesterol-lowering effect of high-molecular-weight β-glucan than TT carriers. The main proposed underlying mechanism is the interruption of bile acid metabolism. No effect of the APOE variants was observed in this study, although the sample size is small [61]. The combined effect of this SNP with variants in genes associated with cholesterol metabolism (rs6720173 and rs760241) affected cholesterol levels in response to dairy product consumption [54]. Interestingly, these findings suggest that the rs3808607 variant may modulate the effect of dietary interventions with compounds (soluble fiber and dairy fat) that are expected to affect cholesterol metabolism through different mechanisms.

The F*ADS1* and *FADS2* genes (encoding for fatty acid desaturases 1 and 2, respectively) have multiple SNPs. The variant rs174550 in *FADS1* is an intronic variant in the gene encoding the enzyme that converts linoleic acid (LA) into arachidonic acid (AA), and the effect of the SNP on the protein expression or function is unknown. A study showed that this SNP had no effect in the blood lipid response to the dietary intake of LA and linolenic acid (ALA) [20]. The PUFA-induced Lp(a)-lowering effects are not modified by the FADS1 rs174550 genotype. In the study of Lankinen et al. [18], the rs174550 variant showed genotype by diet interactions modifying proportions of circulating PUFAs in response to an 8-week high-LA diet. It is well known that this genotype modifies proportions of PUFAs in plasma lipids. According to the study conducted by Lankinen et al., 2021, this genotype exerted differential effects on C reactive protein (CRP) plasma levels after consumption of LA [22], although the mechanism is poorly understood [70].

The cholesterol ester transport protein (*CETP*) is a transporter of cholesteryl esters from HDL to lower-density apoB-containing lipoproteins. At the same time, triglyceride is transferred in the opposite direction to HDL. This gene contains polymorphisms with significant interactions with dietary fat. The SNP rs3764261 in the *CEPT* gene has been associated with CVD risk factors and has shown significant interactions with the dietary pattern (Mediterranean diet) modifying HDL-C (*p* = 0.006) and triglyceride concentrations (*p* = 0.040) [35]. The effect of the variant rs5882 in *CETP* was tested as a modifier of the response to the consumption of high-oleic canola oil (HOCO) and HOCO-DHA oil. This combined treatment decreased TG levels by 24% compared to HOCO. There were not significant interactions affecting lipid levels due to the DHA supplementation [23].

The gene *LIPC* encodes for the enzyme hepatic lipase, which is produced in the liver and released to the bloodstream. Its function is to hydrolyze TG and phospholipids from lipoproteins (Table 4). SNPs in this gene have shown interactions with dietary compounds in cross-sectional studies. In the study by Smith et al. [49], the major allele carriers (CC/CT) of rs1800588 variant had higher HDL-C following the Western diet, rich in saturated fat, compared with the Hispanic diet, containing MUFAs. In contrast, HDL-C levels in individuals carrying the TT genotype did not differ by diet. Dietary fat intake modifies the effect of the variant rs2070895 in LIPC on changes in serum lipids during a long-term weight-loss intervention in adults with obesity [51]. This SNP is in high linkage disequilibrium with rs1800588. These findings suggest that variants in this gene increase circulating HDL-C in response to the amount of total dietary fat.

The interactions of SNP within other genes relevant for CVD, such as the *ATP binding cassete A member 1 (ABCA1)*, *ATP-binding cassette transporter G5 and G8 (ABCG5*, *ABCG8)* [19,21], apolipoprotein A1, APOA1 [71], apolipoprotein A5, APOA5 [72], have been analyzed in a smaller number of studies that fulfilled the inclusion criteria and have shown less consistent effects on CVD risk factors. These genotypes influence changes in blood lipids in response to modifications of dietary fat. In the case of variants within the peroxisome proliferated activated receptors (PPARs) alfa and gamma genes, four studies were found showing significant interactions with the diet [23,25,65,73]. The role of common SNPs in these genes on the response to the intake of different fatty acids has been tested showing that the intake of omega-3 PUFAs exerts different effects on triglycerides and insulin metabolism, according to PPARs genotype [73]. In contrast, a study testing the interaction between the intake of milk with different fat content and rs135549 in PPARα showed no effect on CVD risk factors, probably due to the limited effect of saturated fat on this transcription factor [65].

Variants in enzymes in the folic acid metabolism affect homocysteine levels. The enzyme methylenetetrahydrofolate reductase (*MTHFR*) is an enzyme in the folate metabolism necessary for one-carbon metabolism and production of nucleic acids. Studies on the variants in enzymes of the mentioned pathway analyzed the effect of supplementation with B vitamins on the levels of blood homocysteine and LDL-C, showing a significant reduction in the treatment group as compared to placebo [44]. A similar genotype effect was observed in patients with hemodialysis supplemented with folic acid and vitamin B12 [45]. The variant rs1076991 C > T in the gene methylenetetrahydrofolate dehydrogenase (MTHFD1) is associated with increased risk of acute myocardial infraction (AMI), although the risk seems to be dependent on specific vitamin B supplementation [46]. This variant exerts a significant effect on the promoter activity of this gene.

### 4.2. Effects of Interventions

The modification of the amount and type of fat is a well-known factor to induce changes in circulating lipids and other metabolic parameters. The interventions testing the effects of dietary lipids analyzed the effects of decreasing total dietary fat and the substitution of saturated fat by MUFAs and PUFAs.

The effects of the dietary MUFAs and PUFAs on biomarkers for CVD have been demonstrated [24,25,27,28]. The intake of fatty acids change the composition of cell membranes, influencing fluidity, intracellular signaling, gene expression and availability of substrates for lipid-derived inflammatory regulators [74]. Diets containing MUFAs produce larger and less atherogenic chylomicrons compared with diets with saturated fat; this difference may explain the association of consumption of MUFAs with a decrease in the risk for CVD [75]. Differences in the metabolic effects between omega-3 and omega-6 PUFAs have been thoroughly analyzed. In the study by Nuotio et al., 2024, the omega-3 alfa-linolenic acid (ALA) appears to decrease LDL-C, non-HDL-C, remnant-C and ApoB more effectively than omega-6 linoleic acid (LA) [18]. Omega-3 fatty acids reduce TG and inflammation and may modify insulin sensitivity. In addition, differential effects of omega-3 and omega-6 PUFAs on lipoprotein characteristics have been reported [75]. Some of the effects of omega-3 PUFAs are mediated by the activation of the PPAR transcription factors that increase oxidation of fatty acids, decrease VLDL, and may improve insulin sensitivity [25,74].

The Mediterranean dietary pattern (Table 2) is characterized by a high content of fruits, vegetables, whole grains, and healthy fats and has been consistently associated with better cardiovascular health. Thus, it combines the effects of a group of bioactive compounds such as fatty acids, fibers, and phytochemicals [76]. According to the present review, this dietary pattern may reduce TG, total cholesterol, and inflammation-related phenotypes, and these effects may vary according to genotypes in lipid metabolism. Interestingly, a study found an effect of the C677T (rs1801133) variant in *MTHFR* on the response to the Italian Mediterranean diet (IMD) and the Italian Mediterranean organic diet (IMOD). A significant interaction between this genotype and the diet on homocysteine levels was found when compared to a low protein diet (*p* < 0.001) in patients with chronic kidney disease and stable renal function [77].

The studies using vitamins (B12, B6, folate) as intervention showed genotype-depending effects on homocysteine concentrations, particularly in participants with variants in the *MTHFR* gene. The rs1801133 SNP in this enzyme is known to decrease the activity. These studies suggest that patients with genetic susceptibility may benefit from a personalized treatment [44,45,46] (Table 3).

The hypocaloric diets (Table 5) with different macronutrient compositions (high protein, low fat) had effects on weight loss and lipid metabolism, modifying the risk for CVD. The observed effects on cardiometabolic markers are very likely influenced by changes in body composition primarily and, to a lesser extent yet in some cases significant, to the effects of specific genotypes.

As observed in Table 6, studies using interventions that included the intake of foods, drinks, or isolated compounds achieved some changes in CVD risk factors. Studies using fiber in the form of oatmeal, glucans, kiwi fruit and avocado reduced TG, total cholesterol, and LDL-C and increased HDL-C. These effects were mediated by genotypes in lipid-related genotypes such as rs708272 in *CETP* and rs3808607 in *CYP7A*1 and *APOE*. The study by Hannon et al. (Table 1) used an intervention with one avocado per day, which combines the effects of fiber and MUFAs in improving dyslipidemia. This study suggests that the intake of a specific food in isoenergetic diets may exert significant genotype-related effects on CVD risk factors, as observed in the study using Hass avocado [21].

The use of isolated nutrients or bioactive compounds allows for testing their effects in a specific manner, using a controlled dose, removing the effect of other components or the overall caloric intake [29,31,52,55,61,63].

In summary, this review identified studies testing gene–diet interactions discovered or confirmed in human experimental studies, providing valuable scientific evidence on the contribution of genetic variation to individual responses to diet. As mentioned before, there is a large methodological diversity among the selected studies as well as a lack of replication studies, particularly across populations other than Caucasian, that may have different variant frequencies and genetic backgrounds. Other considerations are that most of the studies have been conducted in relatively small samples and the fact that the functional effect of most of the identified variants are unknown. Therefore, new studies with proper statistical power are required, testing the effects of known or novel dietary interventions in order to improve the alternatives for prevention and treatment of CVD in populations around the world.

## 5. Conclusions

Numerous gene–diet interactions influencing the response to dietary interventions for CVD-related factors have been identified in intervention studies conducted in humans. Diverse dietary interventions have shown interactions with genotypes which may contribute to understanding inter-individual variation. In CVD, genetic variants in lipid transport and metabolism have shown significant effects, although variation in genes belonging to different pathways have interesting interactions. Replication and validation of the clinical significance of these effects across different populations are required in order to develop tools for precision nutrition.

## Figures and Tables

**Table 1:** Fatty acid interventions: Gene by diet interaction in the treatment of cardiovascular disease risk factors.

Study (Reference)	Population Characteristics	Country	Intervention (Dietary, Treatment)	Genotype(s)	Outcome	Main Findings
Nuotio et al., 2024. [18]	118 participants (METSIM study) Age: 65.9 ± 5.6 y BMI: 24.7 ± 2.6 kg/m^2^ Sex: 100% men	Caucasian	8 weeks of dietary intervention enriched with: Option 1. Camelina sativa oil (ALA diet) or Option 2. Sunflower oil (LA diet) Both: 30–50 mL/day	*FADS1* rs174550	Lp(a), LDL-C, HDL-C and apo-B, levels	The ALA diet lowered serum Lp(a) levels by 7.3% (*p* = 0.003) and LA diet by 9.5% (*p* = 0.089). Both diets led to greater decreases in individuals with higher baseline Lp(a) concentration. LDL-C, non-HDL-C and apo-B were lowered by the ALA diet (*p* < 0.01). Lipid or lipoprotein responses were not modified by *FADS1* rs174550 genotype (*p* > 0.05).
Rajendiran et al., 2021. [19]	92 participants Age: 18 to 65 y Sex: 53% women, 47% men	Canada	4 weeks of intervention with 5 isoenergetic diets, each, with ≥24 day washout periods. Four diets provided 32% energy from fat, with (i) SFAs from cheese, (ii) SFAs from butter, (iii) n-9 MUFAs, and (iv) n-6 PUFAs, (v) lower in fat (25% energy from fat) and higher in carbohydrates.	*ABCA1* rs2230808, rs2066714 *ABCG5* rs6720173 *ABCA5* rs11887534 *APOB* rs676210 *APOE* rs7412, rs429358 *CYP7A1* rs3808607 *DHCR7* rs760241, rs1044482 *LDLR* rs688 *LIPC* rs6083 *LIPG* rs2000813 *LPL* rs3200218 *MTTP* rs2306986 *NPC1L1* rs2073547 *PCSK9* rs562556 *PPARA* rs6008259 *SCAP* rs12487736 *SREBF2*, rs2228314, rs2228313 *LIPA* rs1051338	LDL-C and TG levels	LDL-C was reduced significantly deppending on the source of SFAs (cheese: 3.18 ± 0.04, butter: 3.31 ± 0.04, MUFAs: 3.00 ± 0.04, PUFAs: 2.81 ± 0.04, carbohydrates: 3.11 ± 0.04 mmol/L, all with *p* < 0.001) while TG levels were not significant (*p* = 0.117). On 22 candidate SNPs, only *ABCA1*-rs2066714 and *APOE* isoforms exhibited consistent effects related with LDL-C levels. Therefore, the combinations of SNPs associated with a significant part of the variability in LDL-C and TG concentrations following dietary interventions differing in their fatty acid profiles.
Lankinen et al., 2021. [20]	130 participants (METSIM study) Age: 65.7 ± 5.6 y BMI 24.7 ± 2.6 kg/m^2^ Sex: 100% men	Finland	8 weeks of dietary intervention with LA or ALA-enriched diet (30–50 mL/day). Source of LA: sunflower oil. Source of ALA: *Camelina sativa* oil.	*FADS1* rs174550	Markers of low-grade inflammation and glucose–insulin homeostasis	The sun flower oil dietary group, there is a significant genotype x diet interaction for the proportion of arachidonic acid in plasma phospholipids (*p* < 0.001) and serum hs-CRP (*p* = 0.029). In the C*amelina sativa* oil dietary group, there are significant genotype × diet interactions for n-3 PUFAs, but not for clinical characteristics.
Hannon et al., 2020. [21]	115 participants (PATH study) Age: 35.7 ± 0.6 y BMI: 33.1 ± 0.6 kg/m^2^ Sex: 63% women, 37% men	White, Asian, African American	12 weeks Intervention: Isocaloric meal + Hass avocado (175 g for males, 140 g for females). Control: meal higher in saturated fat and low in fiber.	17 SNPs in10 genes related to lipoprotein metabolism: *CETP*, *ABCA1*, *ANGPTL3/4*, *CD36*, *LPL*, *APOE*, *APOA5*, *GCKR*, *LIPC*, *MLXIPL*.	Total cholesterol, HDL-C, TG, body composition	The avocado consumption may help manage dyslipidemia in adults with overweight and obesity. Three SNP–diet interactions were associated with final TC concentrations: *ANGPTL3*-rs10889337 (*p* = 0.01), *ANGPTL4*-rs2278236 (*p* = 0.02), and *CD36*-rs10499859 (*p* = 0.01). SNPs in *GCKR* and *LPL* were associated with TC changes (*p* = 0.01). The interaction between *GCKR*-rs1260326 and diet was such that C-homozygotes receiving avocado had final TC concentrations that were significantly lower than the C-homozygotes in the control group (*p* = 0.02).
Lankinen et al., 2019. [22]	59 participants (METSIM study) Age: 55 ± 5.6 y BMI: 26.5 ± 3.5 kg/m^2^ Sex: 100% men	Finland	4 weeks of dietary intervention of habitual diet with a supplement of 30, 40, or 50 mL of sunflower oil (62% of LA) daily.	*FADS1* rs174550	Plasma lipids, glucose, and CRP	Responses in concentrations of serum hs-CRP, glucose and the proportion of LA in plasma phospholipids and cholesteryl esters differed between genotype groups (interaction of diet × genotype (*p* < 0.05).
AbuMweis et al., 2018. [23]	130 participants (COMIT study) Age: 46.5 y BMI: ≤29.8 kg/m^2^ Sex: 54% women 46% men	Canadian	4 weeks of intervention each. Phase 1. High-oleic canola oil (HOCO). Phase 2. High-oleic canola oil supplemented with DHA (HOCO-DHA).	*FADS1*, rs174561 *FADS2*, rs174583 *ELOVL2*, rs953413 *ELOVL5*, rs2397142 *CETP*, rs5882 *SCD1*, rs2234970 *PPARα*, rs6008259 *LIPF*, rs814628	Lipid levels	The consumption of HOCO-DHA oil reduced TG levels by 24% compared to HOCO (*p* < 0.05). There were not significant treatment-gene interactions in lipids levels to DHA supplementation (*p* > 0.05). There were not association between lipid levels and any genetic variations (*p* > 0.05).
Takeuchi et al., 2018. [24]	53 participants Age: 40.0 ± 11.8 y BMI: 21.8 ± 3.0 kg/m^2^ Sex: 80% women, 20% men	Japan	4 weeks of intervention with consuming one cookie/day that contained: Option 1: High oleic sunflower oil (control group): Option 2. Partially hydrogenated rapeseed oil (TFA group).	*FTO* rs9939609 *Beta-3 adrenergic receptor* rs4994	Lipid levels	No significant differences in serum cholesterol (total, LDL-C and HDL-C) or TG between the control and trans fatty acid groups were found (*p* > 0.05). The responses of serum lipid levels, glucose, insulin and hemoglobin A1c were also independent of the gene variants.
Binia et al., 2017. [25]	191 participants Age: 26.6 ± 6.3 y BMI: 23.7 ± 2.6 kg/m^2^ Sex: 64% women, 36% men	México	6 weeks of dietary intervention with 3 capsules of fish oil (each 647 mg EPA and 253 mg DHA).	*PPARα* L162V *PPARγ* P12A	Lipid levels and inflammation biomarkers	Carriers of the minor alleles of *PPARα* L162V and *PPARγ2* P12A had larger responses in reduction in TG (*p* = 0.02 and *p* = 0.025, respectively). It was not observe any significant change after intervention in total cholesterol, LDL-C and HDL-C (*p* > 0.05).
Fallaize et al., 2017. [26]	442 participants (LIPGENE study) Age: 55 ± 1 y BMI: 31.6 ± 1.0 kg/m^2^ Sex: 56% women, 44%men	EU, Ireland, UK, Norway, France, Spain, Poland, Sweden	12 weeks of dietary intervention with isoenergetic diets that differed according to fatty acids: (1) HF SFA-rich, (2) HF MUFA-rich, (3) LF high-complex carbohydrate supple-mented with LC n-3 PUFAs and (4) Low-fat high-complex carbo-hydrate supplemented with high-oleic acid sunflower oil.	*APOE* *rs7412* *rs429358*	Plasma fatty acids, blood pressure, insulin sensitivity and lipid concentrations	Fatty acids-*APOE* gene interactions at baseline and following change in plasma FA were assessed (*p* < 0.05). At baseline E4 carriers had higher total cholesterol, LDL-C and apo-B compared with E2 carriers (*p* ≤ 0.001); and higher total cholesterol, LDL-C and apo-B compared with E3/E3 (*p* ≤ 0.001). PUFAs dietary were associated with a beneficially lower concentration of apo CIII in E2 carriers, a high proportion of C16:0 was associated with insulin resistance in E4 carriers (*p* ≤ 0.001).
Shatwan et al., 2017. [27]	120 participants (DIVAS study) Age: 47 ± 9 y BMI: 26.3 ± 3.9 kg/m^2^ Sex: 55% women, 45% men	United Kingdom	16 weeks of dietary intervention with 3 diets (%Total energy derived from SFAs:MUFAs:n-6 PUFAs). (1) rich in SFAs (17:11:4), (2) rich in MUFAs (9:19:4) and (3) rich in n-6 PUFAs (9:13:10).	*APOE* rs405509 rs1160985 rs769450 rs439401 rs445925 rs405697 rs1064725 *LP*L, rs320 rs328	Fasting lipid levels	After the 16 weeks of intervention, a significant diet x gene interaction was observed for changes in fasting total cholesterol (*p* = 0.001). For the *APOE* rs1064725, only TT homozygotes showed a significant reduction in total cholesterol after the MUFA diet compared to other dietary intervention (*p* = 0.004).
Minihane et al., 2016. [28]	312 participants Age: 45 ± 13 y BMI: 25.2 ± 3.4 kg/m^2^ Sex: 52.2% women, 47.8% men	United Kingdom	8 weeks of dietary intervention with oil or fish oil providing 0.7 or 1.8 g EPA + DHA/day.	*eNOS* rs1799983	Blood pressure	No effects of n-3 fatty acid treatment or any treatment x eNOS genotype interactions were evident in the group as a whole for any of the clinical or biochemical outcomes (*p* > 0.05). However, adults with isolated systolic hyper-tension, daily doses of EPA + DHA as low as 0.7 g can bring about clinically meaningful reductions in BP (*p* = 0.046).
Tremblay et al., 2015. [29]	208 participants Age: 30.8 ± 8.7 y BMI: 27.8 ± 3.7 kg/m^2^ Sex: 54% women, 46% men	Canada	6 weeks of dietary intervention with 3 g/day of n-3 PUFAs (5 capsules/day of oil fish containing 1.9 g EPA and 1.1 g DHA).	61 SNPs in *PLA2G2A* P*LA2G2* *PLA2G2D PLA2G2F PLA2G4A* *PLA2G6* *PLA2G7*	TG levels	The n-3 PUFAs supplementation had an independent effect on plasma TG levels (*p* < 0.0001). Genotype effects on plasma TG levels were observed for rs2301475 in *PLA2G2C*, rs818571 in *PLA2G2F*, and rs1569480 in *PLA2G4A*. Genotype x supplementation interaction effects on plasma TG levels were observed for rs1805018 in *PLA2G7* (*p* = 0.0286) as well as for rs10752979 (*p* = 0.0273), rs10737277 (*p* = 0.0241), rs7540602 (*p* = 0.0344), and rs3820185 (*p* = 0.0231) in *PLA2G4A*.
Tremblay et al., 2015. [30]	191 participants (QUSAbec study) Age: 18–50 y BMI: 27.63 ± 3.55 kg/m^2^ Sex: 50% women, 50% men	Canada	6 weeks of dietary intervention with 3 g/day of n-3 PUFAs (5 capsules/day of oil fish containing 1.9 g EPA and 1.1 g DHA).	22 SNPs in *PLA2G4A* 5 SNPs in *PLA2G6*	Total n-6 fatty acids and CRP levels	Supplementation decreased total n-6 FAs without affecting plasma CRP levels. Changes in CRP levels correlated positively with changes in total n-6 FAs in men (r = 0.25 *p* = 0.01), but not in women.
Bouchard-Mercier et al., 2014. [31]	201 participants Age: 30.9 ± 8.7 y BMI: 27.6 ± 3.5 kg/m^2^ Sex: 46% women, 54% men	Canada	6 weeks of intervention with fish oil supplementation (5 g/day: 1.9–2.2 g EPA and 1.1 g DHA).	*SREBF1* rs12953299 rs4925118 rs4925115	Insulin sensitivity	The three SNPs (rs12953299, rs4925118 and rs4925115) were associated with differences in the response of plasma insulin levels (*p* = 0.01, *p* = 0.005 and *p* = 0.004, respectively) and rs12953299 as well as rs4925115 were associated with the insulin sensitivity response (*p* = 0.009 and *p* = 0.01, respectively) to the fish oil supplementation.
Wu et al., 2014. [32]	84 participants Age: 47.6 ± 1.3 y BMI: 26.1 ± 0.5 kg/m^2^ Sex: not reported	United Kingdom	8 weeks of fish oil supplementation with 3 capsules fish oil/day (providing a total daily dose of 0.9 g EPA + 0.6 g DHA) or placebo caps.	*eNOS* Asp298	Lipid levels and inflammatory markers	There was not a significant effect of fish oil n-3 PUFAs, *p* = 0.069. GT/TT subjects tended to have higher concentrations of total cholesterol and LDL-C, but vascular function was not affected by either treatment or *eNOS* genotype.

*ABCA1*, ATP-binding cassette subfamily A, member 1; *ABCA5*, ATP-binding cassette subfamily A, member 5; *ABCG5*, ATP-binding cassette subfamily G, member 5; ADBR2, adrenergic receptor 2; ALA, alfa-linolenic acid; *ANGPTL3/4*, angiopoietin like protein 3 and 4; APOA, apolipoprotein A; *APOB*, apolipoprotein B; APOE, apolipoprotein E; BMI, body mass index; CETP, cholesterol esther transport protein; *CYP7A1*, cholesterol 7α-hydroxylase; *DHCR7*, 7-dehydocholesterol reductase; eNOS, endothelial nitric oxide synthese, FADs, fatty acid desaturase; LA linoleic acid; *LDLR*, LDL receptor; *LIPA*, lipase A, lysosomal acid type; *LIPC*, lipase C, hepatic type; *LIPG*, lipase G, endothelial type; *PPARα*, peroxisome proliferator activated receptor alpha, *PPARγ*, peroxisome proliferator activated receptor gamma, PUFAs, polyunsaturated fatty acids; TG, triglycerides; TNF, tumor necrosis factor.

**Table 2:** Dietary pattern interventions: Gene by diet interaction in the treatment of cardiovascular disease risk factors.

Study (Reference)	Population Characteristics	Country	Intervention (Dietary, Treatment)	Genotype(s)	Outcome	Main Findings
Lewis et al., 2023. [33]	63 participants Age: 56 ± 10 y BMI: 27.6 ± 5.4 kg/m^2^ Sex: 54% women, 46% men	Greenland	4 weeks of dietary interventions. Diet 1. Traditional (marine-based, low-carbohydrate). Diet 2. Western (high in imported meats and carbohydrates).	*TBC1D4* p.Arg684Ter	Glucose tolerance and cardiometabolic markers.	The traditional diet reduced the mean daily blood glucose respect to Western diet (*p* = 0.006). Furthermore, it gave rise to a weight loss of 0.5 kg (*p* = 0.016) relative to the Western diet and 4% lower LDL:HDL ratio, which appeared to be driven by HDL-C elevation (*p* = 0.06). A diet–gene interaction was indicated on insulin sensitivity (*p* = 0.093), which reflected a non-significant increase of 1.4 mmol/L in carrier.
Corella et al., 2018. [34]	7170 participants Age: 67.06 ± 6.2 y BMI: 29.83 ± 3.8 kg/m^2^ Sex: 57% women, 43% men	Mediterranean	7 years of dietary intervention, secondary analysis of PREDIMED diet. Two groups with a Mediterranean diet vegetables intake and control diet	*OGG1* rs1052133 (Ser326Cys)	CVD mortality	A significant protective interactions for CVD mortality was found for vegetable intake (HR ratio interaction; *p* = 0.046). Cys326Cys carriers presented higher total mortality rates than Ser326-carriers (*p* = 0.009).
García-Rios et al., 2018. [35]	424 participants Age: 59.46 ± 0.3 y BMI: 30.93 ± 0.13 kg/m^2^ Sex: 13% women, 87% men	Spain	12 months intervention with Mediterranean diet (35% fat, 22% MUFA) vs. Low-fat diet (28% fat, 12% MUFA).	*CETP* rs3764261	Plasma lipid concentration	A significant gene–diet interaction between rs3764261 and the dietary pattern for HDL-C (*p* = 0.006) and TG (*p* = 0.040) was found. T carriers had higher plasma HDL-C (*p* = 0.021) and lower TG (*p* = 0.020) compared with GG.
Roncero-Ramos et al., 2018. [36]	1002 participants (CORDIOPREV study) Age: 20 to 75 y BMI: 31.1 ± 0.3 kg/m^2^ Sex: 17% women, 83% men	Spain	7 years of dietary intervention with two diets: Diet 1: Low-fat diet (<30% total fat and a minimum of 55% carbohydrates). Diet 2: Mediterranean diet (35% calories as fat, 15% proteins and 50% carbohydrates).	*NLRP3* rs4612666 rs10754558 rs35829419 rs4353135 rs10733113	Glucose homeostasis and lipid levels	CT + TT-rs4612666 and AG + AA-rs10733113 carriers of *NLRP3* increased insulin sensitivity index (ISI) after three years of dietary intervention (*p* < 0.001). Further analysis by diet showed that the improvement of the ISI in non-diabetic rs10733113 AG + AA carriers was specific to the consumption of the Mediterranean diet (*p* = 0.041).
Corella et al., 2016. [37]	7098 participants Age: 67 ± 6.2 y BMI: 30 ± 3.8 kg/m^2^ Sex: 57% women, 43% men	Spain	4.8 years follow-up of MedDiet intervention with MUFAs from: (1) extra virgin olive oil, (2) Mixed nuts, (3) low-fat diet (Control).	*CLOCK* rs4580704	CVD risk factors and biochemical profile	A significant interaction (*p* = 0.018) between *CLOCK*-rs4580704 and T2D status on stroke. Thus, only in T2D subjects was *CLOCK*-rs4580704 associated with stroke risk, G-carriers having decreased risk (HR: 0.61; *p* = 0.024 vs. CC) in the multivariable-adjusted model.
Gómez-Delgado et al., 2015. [38]	897 participants (CORDIOPREV study) Age: 59.48 ± 4.48 y BMI: 31.19 ± 0.22 kg/m^2^	Spain	12 months of dietary intervention with: Diet low-fat (LF) or Mediterranean diet (MedDiet)	*CLOCK* rs1801260 rs3749474 rs4580704	Lipid metabolism and inflammation status	A significant gene–diet interactions between *rs4580704-C/C* and the LF diet shown a decrease in high sensitivity CRP (*p* < 0.001) and an increase in HDL/apo-A1 ratio (*p* = 0.029) than minor G allele carriers. No other gene–diet interactions were observed in this research.
Kang et al., 2015. [39]	93 participants Age: 50.33 ± 1.40 y BMI: 25.5 ± 0.35 kg/m^2^ Sex: 79% women, 21% men	Korea	12 weeks of dietary intervention replacing of refined rice intake with one third legumes, one third barleys, and one third whole grains, 3 times/day, and increased vegetable intake to at least six units (30 to 70 g/unit)/day for sufficient dietary fiber intake.	*APOA5* -1131C	apoA-5 and TG levels	*APOA5*-1131C variant modulated the effect of carbohydrates source influencing TG and apoA-5 levels. In the whole grain and legume group, C-allele carriers showed a significant decrease in insulin (*p* = 0.008), an increase in HDL-C (*p* = 0.020) and apoA-5 (*p* = 0.005); TT allele carriers showed a marginal decrease in insulin (*p* = 0.074) (interaction *p* < 0.001).
Corella D. et al., 2014. [40]	7187 participants (PREDIMED study) Age: 67 ± 6.2 y BMI: 30 ± 3.8 kg/m^2^ Sex: 57% women, 43% men	Europe	3 years of MedDiet intervention rich in: (1) Extra-virgin olive oil, (2) Nuts and (3) Control group (low-fat diet)	*LPL* rs13702	Cardiovascular risk factors	The *LPL*-rs13702 was associated with TG at 3 years (main effects, *p* = 2.1 × 10^−7^). It was detected a significant gene–diet interaction (*p* = 0.025) between this SNP and the dietary intervention for changes in TG. In the entire population, it was observed no significant association between the *LPL*-rs13702 and total CVD (*p* = 0.569).
Di Daniele et al., 2014. [41]	40 participants Age: 46.25 ± 5.97 y BMI: 28.1 ± 2.9 kg/m^2^ Sex: 100% men	Italy	28 days of dietary intervention. 14 days: Italian Mediterranean Diet (IMD). 14 days: Organic diet (IMOD)	*MTHFR* C677T	Homocystein, body composition assessment and biochemical analysis.	A significant interaction between *MTHFR* and the effect of both the IMD and IMOD on homocysteine levels compared to low protein diet (interaction *p* < 0.001). Both the IMD and IMOD resulted in significant variations in anthropometric and laboratory measurements.
Gómez-Delgado et al., 2014. [42]	1002 participants (CARDIOPREV study) Age: 59.9 ± 0.58 y BMI: 32.3 ± 0.29 kg/m^2^ Sex: women and men (% not reported)	Spain	12 months of dietary intervention: (1) MedDiet rich in fat from olive oil (2) Low-fat diet	*TNF* rs1800629 rs1799964	Lipid and glucose metabolism	After 12 months of MedDiet a decreasing in TG and hsCRP was statistically significant in G/G subjects compared with A-carriers (*p* = 0.005 and *p* = 0.034, respectively). No other gene–diet interactions were observed in either diet.
Ortega-Azorín et al., 2014. [43]	7166 participants Age: 66.85 ± 6.3 y BMI: 29.9 ± 3.9 kg/m^2^ Sex: 58% women 42% men	Europe	4.8 years of intervention with: (1) Mediterranean diet rich in extra virgin olive oil and nuts (30 g/day) (2) Control diet	*MLXIPL* rs3812316 C771G Gln241His	Myocardial infarction and lipid levels	*MLXIPL*-rs3812316 (*p* = 3.8 × 10^−6^) and the MedDiet intervention (*p* = 0.030) were significantly associated with decreased TG. Higher adherence to MedDiet increased the protection of lower myocardial infarction incidence in G-carriers versus CC.

*APOA5*, Apolipoprotein A-5; apoA-5, apolipoprotein A-5 levels; apo-A1, apolipoproteína A1; BMI, Body mass index; *CETP*, cholesterol esther transport protein; *CLOCK* Circadian Locomoter Output Cycles Protein Kaput; CRP, C-reactive protein; CVD, Cardiovascular disease; HDL-C, High-density lipoprotein cholesterol; hs-CRP, high-sensitivity C-reactive protein; HR, Hazard Ratios; IMD, Italian Mediterranean diet; IMOD, Italian Mediterranean organic diet; ISI, insulin sensitivity index; LF, Low fat; LDL-C, low-density lipoprotein cholesterol; MUFAs, monounsaturated fatty acid; *MTHFR*, methyltetrahydrofolate reductase; *MLXIPL*, MLX-interacting protein-like, NLRP3, nucleotide-binding domain, leucine-rich-containing family, pyrin domain-containing-3; TBC1D4, TBC1 Domain Family Member 4; TG, triglycerides; TNF, tumor necrosis factor.

**Table 3:** Vitamin interventions: Gene by diet interaction in the treatment of cardiovascular disease risk factors.

Study (Reference)	Population Characteristics	Country	Intervention (Dietary, Treatment)	Genotype(s)	Outcome	Main Findings
Pokushalov et al., 2024. [44]	54 participants Age: 59.2 ± 6.2 y BMI: 24.7 ± 2.6 kg/m^2^ Sex: 58% women, 42% men	Russia	Six months of intervention with 2 caps/day. Placebo or Supplementation of L-methylfolate (1 mg), pyridoxal-5-phosphate (50 mg) and Methylcobalamin (500 mg) per capsule.	*MTHFR* rs1801133 C677T rs1801131 *MTR* rs1805087 *MTRR* rs1801394	Homocysteine and lipid levels	The treatment group had a reduction in homocysteine levels by 30.0% and LDL-C by 7.5% compared to the placebo group (*p* < 0.01). In the subgroup analysis, homozygous minor allele carriers showed a significant reduction in homocysteine (48.3%) and LDL-C (18.6%) levels compared to mixed alleles carriers (*p* < 0.01).
Achour et al., 2016. [45]	132 participants Age: 46.0 ± 15.6 y BMI: Not reported Sex: 60% women, 40% men	Tunisia	6 months of intervention. Group A: 2 months of B9 vitamin (10 mg/day), and 2 months of B12 vitamin (1000 μg) and 2 months of B9 + B12. Group B: 2 months of B12 vitamin, 2 months with folic acid and 2 months with B9 + B12.	*MTHFR* C677T	Total homocysteine (tHcy) Vit B9 and B12 concentrations	In group A, a decreasing in tHcy was found in CC carriers when patients were supplemented with vit B12 only (*p* = 0.009). For CT and TT a significant decrease in tHcy at B9 intake (*p* = 0.038 and *p* = 0.005) and B9 + B12 intake (*p* = 0.024; *p* = 0.017). In group B, CC carriers decreasing tHcy with B9 + B12 intake (*p* = 0.031).
Ding et al., 2016. [46]	2381participants Age: 62 ± 14 y BMI: 26.5 ± 4.6 kg/m^2^ Sex: 21% women, 79% men	Norway	4.9 years intervention with a daily capsule containing one of the following: (1) 0.8 mg folic acid + 0.4 mg vitamin B12 + 40 mg vitamin B6. (2) folic acid + vitamin B12, (3) vitamin B6, (4) Placebo	*MTHFD1* rs1076991	Cardiovascular history and risk factors	An association between the polymorphism and acute myocardial infarction among patients allocated to the combined vitamin B6 and folic acid/B12 treatment (interaction *p* = 0.047 vs. placebo). This interaction seemed to be introduced by a shift from a lower to higher risk of the combined B vitamin treatment according to the number of T allele.

BMI, body mass index; LA linoleic acid; Lp(a) lipoprotein a; *LDLR*, LDL receptor; *LIPA*, lipase A, lysosomal acid type; *LIPC*, lipase C, hepatic type; *LIPG*, lipase G, endothelial type, MTHFR, methyltetrahydrofolate reductase; MTRR, Methyltetrahydrofolate-Homocysteine Methyltransferase Reductase; MTR, 5-Methyltetrahydrofolate-Homocysteine Methyltransferase; tHcy, total homocysteine; TG, triglycerides.

**Table 4:** Macronutrient interventions. Gene by diet interaction in the treatment of cardiovascular disease risk factors.

Study (Reference)	Population Characteristics	Country	Intervention (Dietary, Treatment)	Genotype(s)	Outcome	Main Findings
Sun et al., 2019. [47]	692 participants Age: 51.4 ± 9.0 y BMI: 32.7 ± 3.89 kg/m^2^ Sex: 61% women, 39% men	Caucasian	2 years of dietary intervention with four groups % (fat/protein/carbohydrate): Diet 1: 40/25/35 Diet 2: 20/25/55 Diet 3: 20/15/65 Diet 4: 40/15/45	The BP-polygenic score (BP-PGS) was calculated based on the 66 SNPs from a BP-GWAS 13 SNPs for SBP 12 SNPs for DBP 41 SNPs for both	Blood pressure	Participants in the bottom vs. upper tertile of SBP/DBP-PGS had a greater decrease in SBP (*p* = 0.001) and DBP (*p* < 0.001). Gene–diet interaction had changes in SBP from baseline to 24 months (interaction *p* = 0.009). Participants with high-protein diet had greater decreases in SBP at 6 months (*p* = 0.018), 12 months (*p* = 0.007) and 24 months (*p* = 0.089).
Griffin et al., 2018. [48]	389 participants Age: 51.5 ± 9.5 y BMI: 28.45 ± 4.55 kg/m^2^ Sex: 42% women, 58% men	White Caucasian	4 weeks of diet intervention with isoenergetic variations. Reference dietary: Fat and glycemic index (HSFA/HGI) Diet 1: High MUFAs/low GI (HM/LGI) Diet 2: Low fat (LF)/high GI (LF/HGI) Diet 3: LF/LGI Diet variartion (diet 1–3) was high-MUFAs	*APOE* *E2/3/44*	Plasma lipids and glucose homeostasis index	There was a significant diet x genotype interaction to a decrease total cholesterol (*p* = 0.02) and apo-B (*p* = 0.006) levels among carriers of E4, when SFA was replaced with low GI carbohydrate (*p* = 0.02), and a relative increase in total cholesterol, when SFA was replaced with MUFAs and high GI carbohydrates (*p* = 0.03). Carriers of E2 had an increase in TG when SFA was replaced with MUFAs and low glucose index carbohydrates (*p* = 0.001).
Smith et al., 2017. [49]	42 participants Age: 39.9 ± 10.4 y BMI: 26.9 ± 3.2 y Sex: 75% women, 25% men	USA	4 weeks of dietary intervention with 2 diets of different fat content: Phase (1) High-fat Western diet (39% fat) and Phase (2) Low-fat traditional Hispanic diet (20% fat).	*LIPC* rs1800588	HDL-C, TG, LDL-C, total cholesterol, and OGTT 2 h glucose concentrations	No significant gene–diet interactions were observed for HDL-C (*p* > 0.05). However, *LIPC*-CC/CT carriers increased the HDL-C levels with Western diet compared with the Hispanic diet: phase 1 (*p* = 0.0004); phase 2 (*p* = 0.0003). In contrast, HDL-C in TT individuals did not differ by diet (*p* > 0.05). Only major allele carriers benefited from the higher-fat diet for HDL-C.

APOE, apolipoprotein E; BMI, body mass index; GWAS, Genome-wide association study; HGI, High glycemic index; *HDL-C*, high-density lipoprotein cholesterol *HDL-C*; low-density lipoprotein cholesterol; *LIPC*, lipase C, hepatic type; LGI, Low glycemic index; PUFAs, polyunsaturated fatty acids; SFAs, saturated fatty acids; TGs, triglycerides; TNF, tumor necrosis factor.

**Table 5:** Hypocaloric diet interventions: Gene by diet interaction in the treatment of cardiovascular disease risk factors.

Study (Reference)	Population Characteristics	Country	Intervention (Dietary, Treatment)	Genotype(s)	Outcome	Main Findings
De Luis et al., 2016. [50]	283 participants Age: 52.6 ± 10.8 y BMI: 35.4 ± 6.1 kg/m^2^ Sex: 75% women, 25% men	Spain	9 months of dietary intervention:Diet HP: High protein/low carbohydrate. Diet S: Standard hypocaloric.	*UCP3* rs1800849	Cardiovascular risk factors	With both diets and only in rs1800849-T genotype (diet HP vs. diet S), total cholesterol (*p* < 0.05) and LDL-C (*p* < 0.05) decreased. With diet HP and only in rs1800849-T genotype, glucose (*p* < 0.05), TG (*p* < 0.05), insulin levels (*p* < 0.05) and HOMA-R (*p* < 0.05) decreased.
Xu et al., 2015. [51]	743 aparticipants Age: 50.1 ± 8.9 y BMI: 32.7 ± 3.8 kg/m^2^ Sex: 61% women, 39% men	USA	2 years of dietary intervention in 4 diets, varing % fat, protein, and carbohydrate: (1) 20%, 15%, and 65% (2) 20%, 25%, and 55% (3) 40%, 15%, and 45% (4) 40%, 25%, and 35%	*LIPC* *rs2070895*	Lipid levels	Dietary fat modified effects of the variant on total cholesterol, LDL-C and HDL-C (in teraction, *p* = 0.0008, 0.004, and 0.03, respectively). In the low-fat group, the A-allele was associated with a decrease in total cholesterol and LDL-C (*p* = 0.07 and *p* = 0.06, respectively) and an increase in HDL-C levels (*p* = 0.048), whereas an opposite effect in the high-fat diet group was evident (total cholesterol, *p* = 0.008 and LDL-C, *p* = 0.07).

BMI, body mass index; HDL-C, High-density lipoprotein cholesterol; HOMA-IR, homeostasis model assessment-estimated insulin resistance; HP, High protein; LDL-C, low-density lipoprotein cholesterol; *LIPC*, lipase C; S, Standard; *UCP3*, Uncoupling Protein 3.

**Table 6:** Other nutrient interventions: Gene by diet interaction in the treatment of cardiovascular disease risk factors.

Study (Reference)	Population Characteristics	Country	Intervention (Dietary, Treatment)	Genotype(s)	Outcome	Main Findings
Kopecky et al., 2022. [52]	54 participants Age: 49 ± 12 y BMI: 27.2 ± 3.4 kg/m^2^ Sex: 67% women, 33% men	Canada and USA	12 weeks of interven-tion divided in 3 phases (4 weeks each). Snacks containing cholesterol-lowering bioactive compounds (≥5 g fiber, 1000 mg ω-3 (n-3) fatty acids, 1000 mg phytosterols, and 1800 μmol antioxidant per serving). Control products calorie matched from the grocery marketplace.	*CYP7A1* rs3808607	Total cholesterol, HDL-C, LDL-C, TG, fasting glucose, insulin and CRP levels.	The consumption of the snacks containing a compendium of cholesterol-lowering bioactive compounds reduced the LDL-C (*p* < 0.0001) and TG levels (*p* < 0.0001) compared with control foods. SNPs were not significantly related to outcomes (*p* = 0.230).
Ye, et al., 2020. [53]	56 participants Age: 48.3 ± 3.05 y BMI: 23.2 ± 0.81 kg/m^2^ Sex: 62.5% women, 37.5% men	China	45 days of intervention with staple food substitute. Option 1: 80 g/day oatmeal. Option 2: 80 g/day refined white rice.	CYP7A1 rs3808607 *APOE* rs429358 rs7412	Lipid levels	The only genotype–diet interactions were observed between oat meal consumption and the variant *CYP7A1*-rs3808607 with low LDL-C levels (*p* = 0.04); rs3808607-TT exhibited significantly higher responsiveness to oatmeal (reduction in LDL-C) than G-carriers (*p* = 0.02).
Abdullah et al., 2018. [54]	101 participants Age: 18–69 y BMI: Not reported Sex: 71% women, 29% men	Canada	4 weeks of dietary intervention of dairy products (3 serving/day): 375 mL/day of 1% milk fat (MF), 175 g/day of 1.5% MF creamy stirred fruit-flavored yogurt, and 30 g/day of 34% MF cheddar cheese.	*ABCG5* rs6720173 *CYP7A1*, rs3808607 *DHCR7* rs760241	Lipid and cholesterol levels	There is a combined effect of the variants *ABCG5* rs6720173-C, *CYP7A1* rs3808607-TT, and *DHCR7* rs760241-GG on LDL-C levels following a blended dairy intake (3 servings/day for 4 weeks) intervention (*p* = 0.0016).
Ebrahimi-Mameghani et al., 2018. [55]	80 participants Age: 38.9 ± 6.9 y BMI: 34.3 ± 4.15 kg/m^2^ Sex: 72% women, 28% men	Iran	12 weeks of intervention with Artichoke leaf extract (ALE) supplementation: Group 1. 1800 mg/day of ALE as four tablets. Grupo 2. 1800 mg/day of placebo as four tablets.	*TCF7L2* rs7903146	Anthropometric indices, blood pressure, glucose and lipid profile levels	ALE supplementation decreased insulin level and the HOMA-IR in patients with the of *TCF7L2*-rs7903146-TT polymorphism (*p* < 0.05). There was no significant interaction between blood pressure, glucose, and lipid profile response to ALE supplementation (*p* > 0.05).
Gu et al., 2018. [56]	600 participants Age: 39.5 ± 8.5 y BMI: 23.4 ± 3.24 kg/m^2^ Sex: 56% women, 44% men	China	14 days of dietary intervention: 7 days with low-NaCl diet (3 g NaCl) followed by a 7 days of high-NaCl diet (18 g NaCl)	*SCNN1A* rs11614164 rs4764586 rs3741914 *SCNN1B* and *SCNN1G*	Blood pressure	The common variants in *SCNN1A*, rs11614164, rs4764586, and rs3741914 were associated with salt-sensitivity (*p* = 4.4 × 10^−4^, *p* = 1.1 × 10^−8^, and *p* = 1.3 × 10^−3^). Each copy of the minor allele of rs4764586 was associated with an increased odds of salt-sensitivity, whereas each copy of the minor allele of rs11614164 and rs3741914 was associated with a decreased odds of salt-sensitivity, respectively.
Wang et al., 2017. [57]	30 participants Age: 59 ± 4 y BMI: 28.5 ± 4.8 kg/m^2^ Sex: 60% women, 40% men	Canada	5 weeks of dietary intervention with breakfast rich in barley β-glucan: (1) 3 g of high-molecular weight (HMW), (2) 5 g low-molecular weight (LMW), (3) 3 g LMW or (4) control diet.	*CYP7A1* rs3808607	Cholesterol metabolism and bile acid synthesis	A significant diet x gene interaction was observed for changes in fasting total cholesterol (*p* = 0.001). For the *APOE* rs1064725, only TT homozygotes showed a significant reduction in total cholesterol after the MUFAs diet compared to the SFAs or n-6 PUFAs diets (*p* = 0.004).
Chu et al., 2016. [58]	334 participants Age: 38.4 ± 9.9 y BMI: 22.2 ± 3.1 kg/m^2^ Sex: 47% women, 53% men	China	21 days of intervention: 7 days low NaCl (3 g/day) 7 days high NaCl (18 g/day) 7 days high NaCl + KCl (4.5 g/day)	*ADIPOQ:* rs16861194 rs182052 rs16861205 rs822394 rs12495941 rs2241767 rs2082940	Blood pressure	The rs16861205-*ADIPOQ* was associated with a decreased in DBP to low-NaCl, and an increase in DBP and MAP to high-NaCl (*p* = 0.028, *p* = 0.023 and *p* = 0.027, respectively). The rs822394 was associated with a decreased in DBP and MAP in responses to low-NaCl and an increase in DBP to high-NaCl (*p* = 0.023, 0.030 and 0.033, respectively). An association existed between rs16861194 and SBP in response to KCl intervention (*p* = 0.026).
Gepner et al., 2016. [59]	54 participants Age: 57.1 ± 6.5 y BMI: 29.3 ± 4 kg/m^2^ Sex: 15% women, 85% men	Israel	6 months of dietary intervention with Mediterranean diet and one option: 150 mL/dinner dry red wine or mineral water.	*ADH* Arg48His rs1229984	Blood pressure	A transient decrease in BP was observed in the red wine group at midnight (3–4 h after intake, (*p* = 0.031) and the following morning at 7–9 am (*p* = 0.014). Red wine consumers and homozygous for the gene encoding Arg48His; rs1229984, TT, (fast ethanol metabolizers), exhibited a reduction in 24 h SBP, *p* = 0.002) compared to heterozygotes.
Guo et al., 2016. [60]	1906 participants Age: 38.7 ± 9.6 y BMI: 23.3 ± 3.2 kg/m^2^ Sex: 47% women, 53% men	China	14 days of dietary intervention: 7 days of low sodium (3 g NaCl). Followed by 7 days of high sodium (18 g NaCl).	*NCBT* 76 SNPs	Blood pressure	S*LC4A4*-rs4254735 was associated with a decreased DBP to low NaCl (*p* = 5.05 × 10^−4^). In addition, carriers of rs10022637-C *SLC4A4* with high NaCl intervention increased their SBP (*p* = 1.14 × 10^−4^), DBP (*p* = 2.26 × 10^−5^) and BP (*p* = 2.07 × 10^−6^).
Wang et al., 2016. [61]	30 participants Age: 59 ± 4.5 y BMI: 28.5 ± 2.4 kg/m^2^ Sex: 60% women, 40% men	Canada	20 weeks of dietary intervention divided in 4 phases, each was 5 weeks and separated by ≥4 weeks washout period. β-glucan varied in molecular weight and dose: 3 g HMW/day 3 g LMW/day 5 g LMW/day and Wheat and rice (WR) control.	*CYP7A1* rs3808607 *APOE* rs429358 rs7412	Cholesterol levels	Consumption of 3 g HMW β-glucan/day lowered total cholesterol compared with the control diet (*p* = 0.0046). This effect was associated with gene–diet interaction, whereby carriers of rs3808607-G allele on *CYP7A1* had greater responses to 3 g HMW β-glucan/day in lowering total cholesterol than TT carriers (*p* = 0.0006). However, LMW β-glucan, at either 3 g/day or 5 g/day did not change cholesterol levels.
MacKay et al., 2015. [62]	71 participants Age: 55.04 ± 8.42 y BMI: 28.12 ± 5.45 kg/m^2^ Sex: 60% women, 40% men	Canada	12 weeks of dietary intervention divided in two 28 days periods and 4 weeks of washout, which the participants consumed their habitual diets. PS period: 2 g PS/day in margarine.	*CYP7A1* rs3808607 *APOE* e3, e4 *CETP* rs5882 *ABCG8* rs4148217	Lipid levels	*CYP7A1*-rs3808607 -T/T showed no LDL-C decrease, while the G-allele was associated with LDL-C response in a dose-dependent fashion (*p* = 0.0006). Similarly, *APOE* e3 carriers (*p* = 0.0370) responded less than *APOE* e4 carriers (*p* = 0.0001). Moreover, genoset *CYP7A1*-rs3808607 T/T/*APOE* e3 was associated with nonresponsiveness (*p* = 0.9999). rs5882 in *CETP* and rs4148217 in *ABCG8* did not associate with LDL-C lowering.
Mackay et al., 2015. [63]	71 participants Age: 55.04 ± 8.42 y BMI: 28.12 ± 5.45 kg/m^2^ Sex: 60% women, 40% men	Canada	12 weeks of dietary intervention divided in two 28 days periods and 4 weeks of washout, which the participants consumed their habitual diets. PS period: 2 g PS/day in margarine.	*CYP7A1* rs3808607 *CETP* *rs5882* *ABCG8* *rs4148217* *APOE* *rs7412* *rs429358*	Lipid levels	*CYP7A1*-rs3808607 T/T showed no LDL-C decrease, while G-allele was associated with LDL-C response in a dose-dependent fashion (G/T, *p* = 0.0006; G/G, *p* = 0.0009). *APOE* ɛ3 carriers (*p* = 0.0370) responded less than *APOE* ɛ4 carriers (*p* < 0.0001). Genotypes are associated with the extent of reduction in circulating LDL-C in response to PS consumption.
Gammon et al 2014. [64]	85 participants Age: 48.5 ± 9.5 y BMI: 27.3 ± 3.95 kg/m^2^ Sex: 100% men	New Zealand	8 weeks of dietary intervention: 4 weeks of healthy diet run-in period before being randomized to one of two 4 weeks intervention sequences of (1) two green kiwi fruit/day plus healthy diet (kiwifruit) (2) Control group: healthy diet	*CETP* rs708272 *APOA1* rs670 *LIPC* rs1800588 *LIPG* rs6507931	Lipid levels	A significant *CETP* Taq1B genotype for intervention was observed for the TAG:HDL-C ratio (*p* = 0.03). B1/B1 homozygotes had a significantly lower TAG:HDL-C (*p* = 0.03) ratio after the kiwi fruit intervention than after the control intervention, whereas the ratio of B2 carriers was not affected. The lipid response was not affected by other gene polymorphisms.
Loria-Kohen et al., 2014. [65]	161 participants Age: 25–65 y BMI: 28.41 ± 0.67 kg/m^2^ Sex: 16% women 84% men	Spain	1 year of dietary intervention with usual diets, adding: (1) 500 mL/day of skimmed (S) milk; (2) Semi-skimmed (SS) milk	*PPARα* rs135549	Cardiovascular risk factors: Total cholesterol HDL-C, LDL-C	*PPARα* rs135549-TT was associated with a reduction in the TC/HDL and LDL/HDL ratios after 12 months of S milk intake (*p* = 0.0015 and *p* = 0.0005, respectively). No differences were observed after consuming either S or SS milk in the C-carriers.

*ABCG5*, ATP-binding cassette subfamily G, member 5; ADIPOQ, Adiponectin, C1Q And Collagen Domain Containing; ADH, Antidiuretic hormone; APOE, apolipoprotein E; BMI, body mass index; CETP, cholesterol esther transport protein; *CYP7A1*, cholesterol 7α-hydroxylase; *DHCR7*, 7-dehydocholesterol reductase; CRP, C-reactive protein; HDL-C, High-density lipoprotein cholesterol; LDL-C, low-density lipoprotein cholesterol; *LIPG*, lipase G, endothelial type; TGs, Triglycerides; TCF7L2, Transcription factor 7 like 2; SCNN1A, Sodium Channel Epithelial 1, A, B and G.

## Data Availability

Not applicable.

## Supplementary Materials

The following supporting information can be downloaded at: https://www.mdpi.com/article/10.3390/healthcare12222292/s1, Preferred Reporting Items for Systematic reviews and Meta-Analysis extension for Scoping Reviews (PRISMA-ScR) Checklist. Reference [78] is cited in the supplementary materials.
